# Occupational Respiratory Diseases of Miners from Two Gold Mines in Ghana

**DOI:** 10.3390/ijerph14030337

**Published:** 2017-03-22

**Authors:** Esther Ayaaba, Yan Li, Jiali Yuan, Chunhui Ni

**Affiliations:** Department of Occupational Medicine and Environmental Health, School of Public Health, Nanjing Medical University, Nanjing 210000, China; lizerta1@yahoo.com (E.A.); Liyan_NJMU@163.com (Y.L.); yjlgogo@sohu.com (J.Y.)

**Keywords:** gold miners, respiratory disorders, asthma, emphysema, bronchitis

## Abstract

*Objective*: This study investigated respiratory disorders among gold miners in Ghana, a sub-Saharan African country. *Material and Methods*: A cross-sectional exploratory design that employed quantitative methods was conducted among 1001 male workers from the Obuasi and Tarkwa mines from December 2015 to April 2016. A total of 1001 workers, consisting of 505 and 496 underground and surface miners, respectively, were involved. The cross-sectional descriptive design was used because data was collected from participants of different experiences by selected participants at a time. *Results*: The study found significant association between age, educational background, marital status and drinking alcohol on respiratory disorders. The prevalence of asthma, pneumonia, bronchitis and emphysema were respectively 47.55%, 14.29%, 9.69% and 5.10%. Coughing was the most cited respiratory symptom (35.4%). *Conclusions:* The study documents important evidence on the level of respiratory disorders among miners in Ghana. Instituting appropriate health education interventions and improving the working environment is critical to improving the overall health and preventing respiratory disorders among miners.

## 1. Introduction

Work-related diseases such as respiratory disorders cause huge suffering and losses in the world of work. However they remain largely invisible in comparison to industrial accidents, although they kill six times as many people annually [1]. Furthermore, the nature of occupational diseases is changing fast as technological and social changes with its concomitant global economic conditions aggravating already existing health hazards and creating new ones. Occupational as well as environmental pollution in the form of dusts, fumes, vapours, and toxic gases are known risk factors for respiratory disorders [2]. The occurrence and severity of mining-related occupational respiratory diseases are said to be a function of the commodities mined, the length of occupational exposure, the levels of occupational exposure, with its concomitant diseases, environmental conditions, and individual’s lifestyle [3].

According to the International Labour Organisation (ILO), an estimated 2.34 million people die every twelve months from work-related accidents and diseases including mine workers. Of these, the vast majority, 2.02 million die from work-related diseases. Also, out of the estimated 6300 work-related deaths that happen twenty-four hours, 5500 are caused by various types of work-related diseases. The ILO further estimates that 160 million cases of non-fatal work-related diseases occur annually [4].

Historically, mining has been associated with health problems caused by occupational and environmental exposure to mine waste, particularly in developing countries [5,6]. A study reported that, one group which is at exceptionally high risk of TB is mineral miners [7,8]. Miners in sub-Saharan Africa are said to have greater incidence of TB than any other working population in the world as 3000–7000 per 100,000 miners per year are reported to contract the disease [8,9]. A study conducted in coal mine workers in Tanzania also found increase prevalence of respiratory symptoms, which were influenced by the level of dust exposure [10]. A similar study that focused specifically on workers with high exposure to coal dust in Tanzania also found significantly high prevalence of acute symptoms of dry cough (45.7%), breathlessness (34.8%) and blocked nose (23.9%) and more chronic symptoms of breathlessness (17.0%) as compared to the lower exposure mine workers [11]. A study among ex- and current miners in South Africa also found high prevalence of respiratory symptoms, especially among ex-miners [12]. A cross-sectional study of a working population of black South African gold miners found three distinct pulmonary disorders (silicosis-associated pulmonary dysfunction with dyspnea on effort; chronic airflow limitation) which was related to the duration of underground exposure and a chronic bronchitis symptom complex, which reflected the intensity of dust exposure in the workplace [13].

Other studies conducted in develop countries also shown high prevalence of respiratory disorders and symptoms among miners. The National Study of Coal Workers’ Pneumoconiosis in the United States for instance showed that 35% of the workers employed in coal mines before 1970 had chronic bronchitis (chronic cough and phlegm), 43% had shortness of breath and 42% had wheezing [14]. A study of 1185 underground coal miners by Seixas et al. [15] who started mining from 1970 and later also found 28% prevalence of cough, 32% phlegm, 21% chronic bronchitis, 22% breathlessness and 27% wheezing. Other reported prevalence of respiratory symptoms and disorders includes bronchitis (33%), United States [16]; chronic bronchitis (37%), United Kingdom [17]; breathlessness (77%), chronic cough (47%), chronic phlegm (37%), China [18].

Good data provide a basis for designing an effective prevention strategy for occupational health diseases. However, more than half of all countries up to date do not collect adequate statistics for occupational diseases. There is therefore scarcity of evidence of the extent of respiratory diseases among miners from these settings to prompt and support appropriate heath interventions. This study was conducted to assess the common respiratory diseases as well as its determinants among miners in two mines in Ghana.

## 2. Materials and Methods

### 2.1. Study Design and Setting

A cross-sectional exploratory study that employed quantitative methods was conducted among 1001 male workers from the Obuasi and Tarkwa mines from December 2015 to April 2016. A total of 1001 workers, consisting of 505 and 496 underground and surface miners were involved, respectively. The cross-sectional descriptive design was used because data was collected from participants of different background by selected participants at a time. The inclusion criteria were male miners that have worked at the mines for at least one year and voluntarily consented to participate in the study.

The Obuasi Gold Mine is operated by AngloGold Ashanti Ltd. of South Africa whiles the Tarkwa Gold Mine is operated by the Gold Fields Ltd. of South Africa. The Obuasi Gold Mine is an open-pit and underground gold mine, situated near Obuasi and is one of the top-9 largest gold mines on Earth [19]. Gold mining began at the mine more than 112 years ago, in 1897, when it was known originally as Ashati Mine [20]. In 2008, AngloGold Ashanti’s Ashantiland operations, consisting of Obuasi and the Iduapriem Gold Mine, contributed 11% to the company’s annual production [20]. There are currently 2500 miners working in the mine. Some equipment used for mining opertions at the Obuasi Gold Mines are shuttle cars, transmixer sprays, Shotcrete, Scooptram (a rubber tired, battery or diesel-operated piece of equipment designed for cleaning runways and hauling supplies); scissor lifts (a safe way for workers in underground and surface operations to reach elevated work); scalers (used for taking down loose material from the roof, face and rib in hard rock mining); remix trucks, personnel carriers (used to transport miners and equipment from the surface of the mine to the underground working locations); haul trucks, elevating equipment such as dual-engine wheeled, single-engine wheeled, scrapers, center dump haul units, haul trucks, drills, Nobas UB 1254 and fire trace automatic fire suppression systems.

The Tarkwa Mine is located in south western Ghana about 300 km west of Accra and consists of several open pit operations, one CIL plant, and two heap-leach facilities. As of 30 June 2005, proven and probable reserves at Tarkwa were estimated to be about 417,000 kg of gold and to last until 2025 at current production rates [9]. The mine has 3200 workers currently. Equipment currently in use at the mine include mass excavators, motor graders (mining size e.g., CAT 24H), wheel loaders, track loaders, draglines, cable/hammer tractors, bulldozers, water trucks (giant mining type), scalers, haul trucks and dump trucks.

### 2.2. Data Collection and Instruments

The data for this study were collected with questionnaires, which assessed information on demographic characteristics and work history as well as prevalence of respiratory diseases and symptoms. Study participants were selected through a systematic random sampling, where a sampling interval, *k,* was calculated based on the number of workers and the required sample size per mining company. All the questionnaires were self-administered under the guidance of investigators. Investigators further cross-checked the questionnaires in the presence of participants for any errors and insured the completeness and accuracy of the questionnaires.

Data on socio-demographic characteristics covered age, smoking, drinking, education level and marital status. The work history and details involved job status, duration of employment, job type, working years and working hours. Cumulative dust exposure was estimated with the duration of employment and number of hours spent at work, taking into consideration the length of the leave period (14 weeks). The average number of hours spent at work for both underground and ground miners was twelve hours.

Smoking refers to at least one cigarette per day on average (or use of other ways to consume the equivalent of tobacco of one cigarettes) and persistent smoking for more than one year. For drinking, only drinking alcohol on holidays means ‘occasional drinking’, drinking alcohol at least once a week means ‘often drinking’ and there was no consideration for the volume of alcohol consumed.

The prevalence of respiratory diseases and symptoms were assessed by asking participants if they have experienced a respiratory or lung disease. The respiratory diseases explored as outcomes were emphysema, pneumonia, asthma and bronchitis. Information on workers’ perceptions of causes of their conditions was also elicited using the questionnaires.

The study protocol was submitted to the Ethics Committee of the School of Medical Sciences, Kwame Nkrumah University of Science and Technology (KNUST), and the Committee on Human Research, Publications and Ethics (CHRPE) at KATH for ethical clearance before the study commenced. Administrative clearance was also sought from management of the various mining companies. Written informed consent for the study was obtained from respondents and all information obtained remained confidential.

### 2.3. Statistical Analysis

Data were entered, cleaned and analysed with SPSS version 22 (IBM Corp., Armonk, NY, USA). Bivariate associations between the explanatory variables and respiratory diseases were explored using Pearson Chi-square test. A logistic regression analysis was also done to further determine the factors influencing respiratory diseases among miners in Ghana. The outcome was the experience of a respiratory disease. Two models were fit; model 1 involved the socio-demographic and lifestyle factors and model 2 was a combination of model 1 and the factors relating to job and environmental exposures. *p* < 0.05 was considered statistically significant.

## 3. Results

### 3.1. Background Characteristics

The study had a response rate of 83.42% (1001 questionnaires received out of 1200 distributed). Table 1 presents the background information of respondents in this study. The median age was 35 years and majority (68.5%) was in the age of 30 to 40 years. Most of them (77.3%) were Christians. More than 80% of the respondents were married and the mean (SD) number of children was 3 (1.4). About 47% had Junior Secondary School education, whereas only 7.5% and 8.4% had Senior Secondary and Tertiary education, respectively. Most of them were Ghanaians (87.7%) and permanent staff (82.8%) whiles 10.7% was temporary staff. Only 2.2% of them smoked cigarettes while a little more than half of them drank alcohol.

The majority of the subjects were working in helping areas (31.3%) whereas 12.1% and 27.4% were working in mining and tunnelling areas, respectively, Table 2. The mean (Standard deviation, SD) duration of work was 13.1 years (6.8) and it ranged from 1 year to 47 years. The mean (SD) duration of hours spent at work was also 12 h (0.6) and it ranged from 2 h to 14 h. About 93.4% and 89.4% were exposed to dust and high/low temperature conditions respectively. Most of the study subjects take their bath at workplace bathrooms before going home and about 83.6% stated that they wash their working clothes themselves. About 95% eat at the workers’ canteen and 83.2% had never experienced flooding at the workplace.

### 3.2. Prevalence of Respiratory Diseases and Symptoms

**Figure 1:**
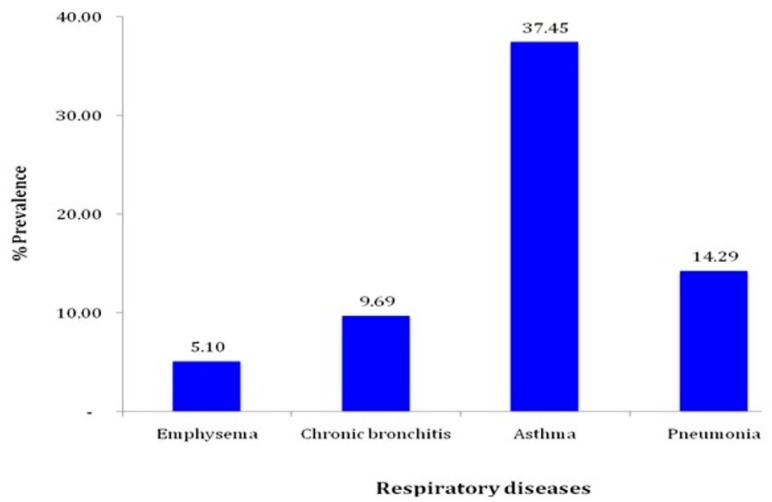
Prevalence of respiratory diseases.

**Figure 2:**
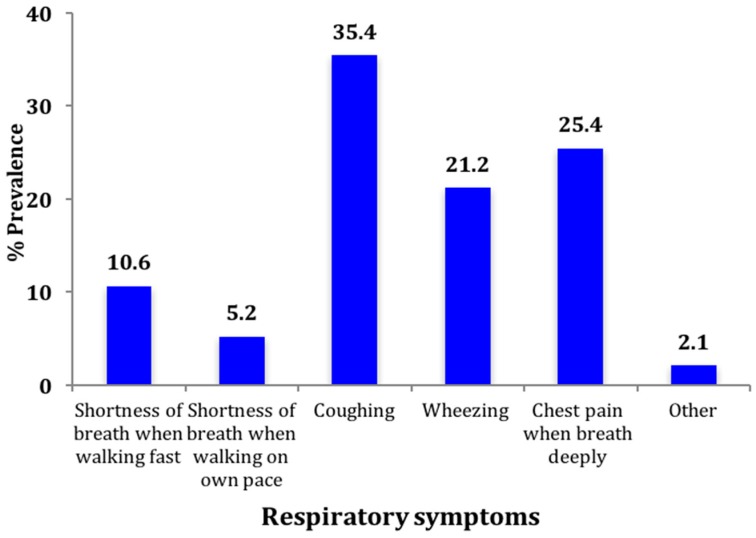
Prevalence of respiratory symptoms (multiple responses).

As shown in Figure 1, asthma was the most cited respiratory disease among the subjects studied (37.45). This was followed by pneumonia (14.29%) and bronchitis (9.69%). The most cited respiratory symptom was coughing (Figure 2).

**Figure 3:**
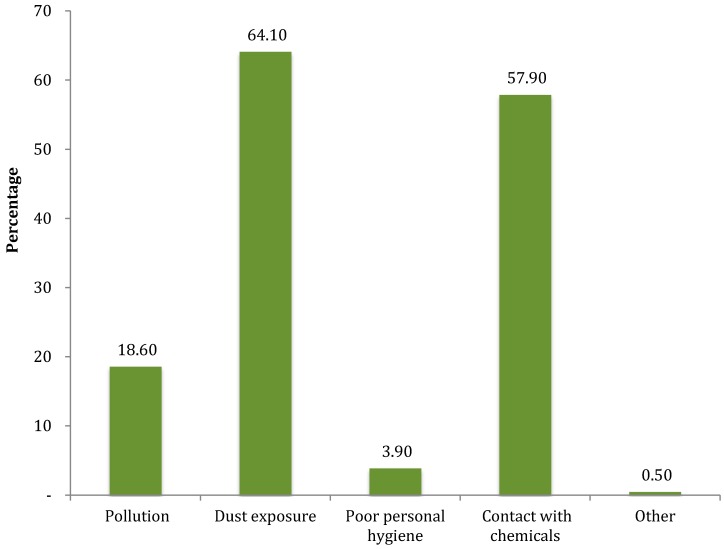
Perceptions about causes of work related illness in the organization (multiple responses).

Other symptoms stated were chest pain (25.4%), wheezing (21.2%) and shortness of breath (10.6%). More than 85% believed that causes of illness in the organization were work-related and the most cited work-related causes were dust exposure (64.1%), contact with chemicals (57.9%) and pollution (18.6%) as shown in Figure 3.

### 3.3. Factors Influencing Respiratory Diseases

Table 3 demonstrates results of bivariate associations between socio-demographic characteristics, work-related factors and respiratory diseases in this study. Age was significantly associated with all respiratory diseases studied. The proportion of subjects with emphysema and pneumonia was higher within the age group of 30 to 40 years whereas bronchitis was highest among those >50 years. Education was also associated with all respiratory diseases with proportion of subjects affected by emphysema and pneumonia being lowest in those with tertiary and diploma education respectively. Smoking was not significantly associated with respiratory diseases whiles drinking alcohol influenced pneumonia (*p* < 0.001), bronchitis (*p* = 0.009) and emphysema (*p* = 0.003).

Table 4 also shows results of bivariate associations between work-related factors and respiratory diseases in this study. The duration of work of respondents was associated with asthma (*p* = 0.027) and pneumonia (*p* < 0.001). The proportion with asthma was higher among those who had worked from 5–10 years (52.2%) and lowest among those who had worked from 10–15 years (25.9%). Cumulative dust exposure was also associated with pneumonia (*p* < 0.001) and emphysema (*p* < 0.001) but not with asthma and silicosis. The proportion of respondents who had pneumonia was significantly higher among those with 10–20 years of cumulative exposure (21.9%) and lower among those exposed for less than 10 years. Working in a dusty environment also influenced the likelihood of being affected by respiratory diseases. Proportions of subjects with respiratory diseases were significantly higher among the exposed than unexposed.

The variables relating to work-related hygiene also showed positive influence on respiratory diseases. Place of bath after work was associated with respiratory disease with the proportion of emphysema and pneumonia being higher among those who bath at the workplace bathroom. Experiencing flooding at the workplace was also significantly associated with asthma (*p* < 0.001), pneumonia (*p* = 0.004) and bronchitis (*p* < 0.001).

### 3.4. Multivariable Logistic Regression

Table 5 presents results of the adjusted odds ratios for factors influencing respiratory diseases among gold miners in Ghana. Model 1 shows results of the influence of socio-demographic factors whiles model 2 involved both the socio-demographic and job and environmental exposures related to mining. Age of the miners was associated with the risk of asthma, pneumonia and emphysema. A year increase in age (years) increased the risk of asthma in the full model (OR; 95% CI = 1.12; 1.06–1.19). An inverse relationship was however observed between age and emphysema, with a 5% decrease in risk with a year increase in age (OR; 95% CI = 0.95; 0.92–0.98). As compared to those who had completed Junior Secondary School education, tertiary education significantly decreased the odds of pneumonia. Marital status of the men also influenced significantly the odds of having emphysema in the model 1 (OR; 95% CI = 0.60; 0.37–0.85) and model 2 (OR; 95%CI = 0.53; 0.34–0.82). The men who drank alcohol also had increased odds of having a respiratory asthma and pneumonia and decreased odds of emphysema.

An increased in the duration of mining also increased the risk of pneumonia (OR; 95% CI = 1.04; 1.00–1.07) and emphysema (OR; 95%CI = 1.04; 1.01–1.06). Having a permanent employment also decreased the risk of asthma, but increased the pneumonia. Miners who worked in combining areas also had significantly increased risk of emphysema as compared to those working at tunneling areas. Working in extreme temperature conditions also increased the risk of respiratory diseases but significance was only observed with pneumonia (OR; 95% CI = 4.62; 1.62–12.96).

## 4. Discussion

Despite the enormous economic benefits associated with mining, its detrimental effects on the environment and health of miners cannot be overlooked. Mining activities, both surface and underground, come along with numerous health externalities and exposure to dust and chemicals from mining causes acute and chronic respiratory diseases. It is hypothesized that this could even be more in limited resource settings, where mining regulations are less enforced and the safety of miners is of less concern. This study was conducted to assess the prevalence of respiratory diseases among miners in Ghana and to further look at the influence of socio-demographic, environmental and work-related factors on respiratory diseases.

This study found a high prevalence of respiratory diseases among gold miners, with asthma being 37.5% followed by pneumonia (14.3%) and bronchitis (9.69%). The prevalence of respiratory symptoms ranged from 35.4% of coughing to 25.4% of chest pain. This corroborates findings from a systematic review of epidemiological studies that linked occupational exposure to dust and respiratory impairment [21]. The prevalence of emphysema in this study was comparable to that reported in a study among South African gold miners, which accessed emphysema using an autopsy database of miners [22]. However, a literature review of the prevalence of Chronic Obstructive Pulmonary Disease (COPD) including emphysema in Africa, reported a high variation in prevalence, ranging from 5.3% to 47% [23].

The prevalence of bronchitis was lower than that reported in an earlier study among underground gold miners in Ghana, 21.2% [24]. There is currently unavailable reliable data to estimate the prevalence of respiratory disease among the general population in Ghana. However, available evidence indicates that the prevalence of chronic bronchitis among the general population in most developing countries could be as high as 27% whereas prevalence among developed countries is estimated to be 3% to 17% on the average [25]. The study by Seixas et al. [15] among underground coal miners in South Africa for instance found a 21% prevalence of chronic bronchitis.

This study also found high prevalence of respiratory symptoms including coughing, chest pain and wheezing. This was consistent with a study among ex- and current miners in South Africa which equally reported high prevalence of respiratory symptoms, especially among ex-miners [12]. Similarly, a study among workers with high exposure to coal dust in Tanzania found significantly high prevalence of acute symptoms of dry cough (45.7%), breathlessness (34.8%) and blocked nose (23.9%) and more chronic symptoms of breathlessness (17.0%) [11]. The prevalence of coughing observed was consistent with findings from a study of coal miners in China, which found a prevalence of 36.3% among miners without Coal Worker Pneumoconiosis (CWP) [26]. 77% of miners in that study had breathlessness walking at a normal pace on level ground whiles 37% had chronic phlegm. Another study of miners exposed to asbestos and coal from different industries in China also found high prevalence of cough, with the prevalence being higher among those with pneumoconiosis [27].

This study further found the influence of socio-demographic and lifestyle characteristics on the prevalence of respiratory diseases among miners in Ghana. Age, education, marital status and alcohol intake were significantly associated with the prevalence of respiratory diseases. A year increase in age was associated with increased risk for asthma and pneumonia but decreased risk for Emphysema among the miners. The inverse relationship between age and emphysema could however be due to the Healthy Worker Effect (HWE), a phenomenon that has been observed in other studies of occupational diseases. This could imply that workers who have been severely ill or chronically disabled are ordinarily excluded from employment [28]. A review of surveillance data for trends by National Institute for Occupational Safety and Health (NIOSH) in 2011 also found that workers at younger ages were already developing coal worker pneumoconiosis [29]. The positive relationship between age and asthma and pneumonia is however consistent with that from the study by Bio in Ghana [24], which found a statistically significant effect of increasing age on prevalence of respiratory problems among miners.

Low level of education was associated with lower risk of respiratory diseases in this study. Miners who had tertiary education were less likely to suffer a respiratory disease as compared to those with only basic education. This corroborates findings from a study in South Africa by Nkosi, Wichmann and Voyi [30], which found lower education to be an independent significant risk factor for chronic respiratory symptoms and diseases. This could be related to job differences and different roles played with respect to one’s level of education. Most underground and labor-intensive staffs in most mining companies in Ghana are indigenes, which mostly have lower educational background. They are therefore more likely to be exposed to dust and other hazardous chemicals that would increase their propensity of acquiring respiratory disease. In many previous studies, lower educational background is associated with higher exposure to hazardous environmental conditions and lifestyles such as smoking that could also lead to respiratory disorders [31]. This association could however be confounded by tuberculosis infection, which is known to be higher among miners with lower educational background. It has also been suggested that individuals with higher levels of education are more likely to have good knowledge of the health effects of environmental exposures, work in a safer environment and report an increased likelihood of having satisfying, personally worthwhile jobs [32,33].

We further found that being married was associated with lower risk of respiratory disorders whereas drinking alcohol increased the risk of respiratory disorders. The unmarried are more likely to lack social support and engage in alcoholism and other lifestyles that make them much prone to respiratory disorders. The influence of alcohol on respiratory infections has been explored in previous studies. Alcohol abuse has been linked to failure to initiate appropriate immune response to pathogens, resulting in lasting effects that alter response to respiratory infections like pneumonia and tuberculosis [34].

Existing evidence have demonstrated a strong relationship between dust and other hazardous environmental exposures and development of respiratory disorders. Exposure to dust is known to produce a variety of clinical responses, including asthma, chronic bronchitis and chronic airways obstructive disease. In line with this evidence, this study found an increased risk of respiratory disease with increasing exposure to dust. A review by Laney and Weissman [35] identified exposure to coal mine to be related to many respiratory diseases and this was attributed to increased levels and duration of dust exposure, increased silica content of dust, and issues related to working in smaller operations. Studies that looked at dust exposures in other working environments also found evidence of inhalation exposure to dust and production of produce allergic respiratory diseases and exacerbation of existing respiratory allergies [36]. A cross-sectional study of a working population of black South African gold miners also found an association pulmonary disorders and the duration and the intensity of dust exposure in the workplace [13]. A study conducted in coal mine workers in Tanzania also found and influence of level of dust exposure as the cause of increased prevalence of respiratory symptoms [10]. Exposure to extreme weather conditions was also associated with development of respiratory disorders in this study. A retrospective analysis to determine the impact of weather on pneumonia mortality in the United States also found mortality from pneumonia to be associated with exposure to periods of below dew point temperature [37]. These findings suggest the need for constant education of miners to ensure appropriate preventive measures to minimize their exposures during work.

The strengths of this study include making available a large quantitative data to allow the investigation into levels of respiratory disorders among miners in Ghana. We have also attempted to investigate and provide evidence of the association between some important socio-demographic characteristics and environmental conditions and the development of respiratory disorders. This study might have some methodological limitations. The choice of cross-sectional design prevents the making of causal claims of the association between workers background and environmental exposures and development of respiratory disorders. The use of questionnaire to investigate miners’ experiences of respiratory disorders other than clinical investigations might also lead to over or underestimation of the prevalence of these conditions. This study was conducted in two mines in Ghana, and the results may be difficult to generalize to other countries, although the information might be valid for the mines elsewhere with similar characteristics. The information obtained will also be useful in improving the working conditions in the mine.

## 5. Conclusions

This study has shown a high prevalence of respiratory disorders among miners in Ghana. This is influenced by their socio-demographic characteristics and environmental exposures at the workplace. In addition to this, Ghana government should develop appropriate training programs for health works on mining related diseases and conduct more research on diseases related to mining occupation.

It is important for mining companies to improve working environment, and continually educate miners on occupational safety measures to help minimize their exposure to hazardous conditions and improve their overall health. Also it is important to note that, occupational health and safety does not only affect the workers and the organization, but also friends, dependents, families, communities and the nation as whole.

## Figures and Tables

**Table 1:** Background characteristics of study subjects.

Variables	Frequency N = 1001	Percentage
Age, years		
<30	149	14.9
30–40	686	68.5
40–50	135	13.5
>50	31	3.1
Median (range)	35 (20–73)	
Religion		
Christianity	774	77.3
Islamic	215	21.5
Traditional/other	12	1.2
Marital status		
Married/living together	854	85.3
Single	137	13.7
Divorced/separated	10	1.0
Number of children		
1	100	10.0
2	145	14.5
3	296	29.6
4 and above	460	45.9
Mean (SD)	3 (1.4)	
Nationality	878	87.7
Ghanaian	21	2.1
Non-Ghanaian	102	10.2
Educational level		
Junior Secondary	466	46.5
Senior Secondary	376	37.6
Diploma	75	7.5
Tertiary	84	8.4
Employment status		
Permanent staff	829	82.8
Temporary staff	107	10.7
Contract	48	4.8
Other	17	1.7
Smoke cigarette	22	2.2
Drink alcohol	534	53.3

SD = Standard deviation.

**Table 2:** Kind of underground mining and working characteristics.

Variables	Frequency	Percentage
Job category		
Tunneling areas	274	27.4
Mining areas	121	12.1
Combining areas	110	11.0
Helping areas	314	31.3
Managerial and others	182	18.2
Duration of work, years; mean (SD)	13.1 (6.8)	
Range	1.0–47.0	
Hours spent at work per day, mean (SD)	11.9 (0.6)	
Range	2.0–14.0	
Worked in environment predisposed to dust	935	93.4
Work in high/low temperature conditions	895	89.4
Place bath before going home after days work		
Bathroom at workplace	837	83.6
Home	164	16.4
Who washes working clothes		
Yourself	853	85.2
Family	123	12.3
Company laundry service	8	0.8
Other	17	1.7
Where eat at work place		
Worker’s canteen	953	95.2
At the work place	46	4.6
Somewhere far off	2	0.2
Experienced flooding while on duty	833	83.2

Tunneling areas: Pneumatic drilling, Blasting or lashing of hard rock materials; Mining area: Cutting and loading; Combining area: Layers for excavation; Helping areas: Maintenance, Transportation, Electromechanical, Equipment.

**Table 3:** Association between background characteristics, work related factors and hygiene on respiratory and lung diseases

Variables	Asthma		Pneumonia		Bronchitis		Emphysema	
	N = 375		N = 143		N = 97		N = 51	
	% Affected	*p*-Value	% Affected	*p*-Value	% Affected	*p*-Value	% Affected	*p*-Value
Age		<0.001		<0.001		0.006		0.001
30	44.3		2.0		14.8		2.7	
30–40	52.9		17.6		7.6		4.2	
40–50	27.4		12.6		12.6		11.9	
>50	32.3		6.5		19.4		6.5	
Educational level		<0.001		<0.001		0.008		0.043
Junior Secondary	48.1		20.6		5.8		6.4	
Senior Secondary	55.6		7.8		12.4		4.0	
Diploma	40.0		5.3		10.7		0.0	
Tertiary	29.8		13.1		11.9		2.4	
Employment status		0.460		0.001		0.197		<0.001
Permanent staff	48.3		16.8		5.4		4.2	
Temporary staff	43.0		3.7		7.5		6.5	
Contract/other	41.7		0.0		0.0		16.1	
Smoking		0.481		0.077		0.173		0.389
Yes	9.1		13.6		18.2		9.1	
No	14.4		5.1		9.5		5.0	
Drink alcohol		0.130		<0.001		0.009		0.003
Yes	44.6	0.130	23.0		7.1		7.3	
No	51.0		4.3		12.7		2.6	

Test = Pearson chi-square.

**Table 4:** Association between works related factors and hygiene on respiratory and lung diseases.

Variables	Asthma		Pneumonia		Bronchitis		Emphysema	
	% Affected	*p*-Value	% Affected	*p*-Value	% Affected	*p*-Value	% Affected	*p*-Value
Job category		0.143		0.837		0.324		0.587
Tunneling areas	45.3		15.3		7.7		5.5	
Mining areas	47.9		14.0		9.9		4.1	
Combining areas	59.1		12.7		7.3		2.7	
Helping areas	46.4		15.3		10.2		6.4	
Managerial and others	45.6		12.1		13.2		4.4	
Duration of work, years		0.027		<0.001		0.120		0.157
<5	39.0		9.1		18.2		9.1	
5–10	52.2		4.9		9.3		5.5	
10–15	25.9		22.2		11.1		3.7	
15–20	47.5		21.7		8.5		3.7	
>20	42.6		11.8		10.3		8.8	
Cumulative dust exposure, years		0.549		<0.001		0.561		
<10	49.8		5.7		10.9		6.2	
10–20	46.4		21.9		8.9		3.4	<0.001
20–30	44.3		11.5		9.8		3.3	
≥30	33.3		11.1		0.0		55.6	
Level of dust exposure		0.001		0.003		<0.001		0.343
High	49.1		15.2	0.003	25.8		7.6	0.343
Low	27.7		1.6		8.6		4.9	
Work in high/low temperature conditions		<0.001		0.071		<0.001		0.012
Yes	50.9		15.0		8.5		5.7	
No	18.9		8.5		19.8		0.0	
Place bath after work		<0.001		<0.001		<0.001		0.193
Bathroom at workplace	50.0		16.5		8.2		5.5	
Home	34.1		3.0		17.1		3.0	
Where eat at work place		0.005		0.018		<0.001		0.258
Worker’s canteen	48.7		13.6		9.1		5.4	
At the work place/Somewhere far off	26.1		28.3		17.4		0.0	
Experienced flooding on duty		<0.001		0.004		<0.001		
Yes	51.1		15.7		8.2		5.3	
No	29.8		7.1		17.3		4.2	

Test = Pearson chi-square.

**Table 5:** Multivariable regression analysis of factors influencing respiratory diseases.

Covariates	Asthma		Pneumonia		Bronchitis		Emphysema	
	Model 1	Model 2	Model 1	Model 2	Model 1	Model 2	Model 1	Model 2
	OR [95% CI]	OR [95% CI]	OR [95% CI]	OR [95% CI]	OR [95% CI]	OR [95% CI]	OR [95% CI]	OR [95% CI]
*Socio-demographic characteristics and lifestyle*								
Age	1.08 [1.04, 1.13] ***	1.12 [1.06, 1.19] ***	1.04 [1.00, 1.08] *	1.03 [0.98, 1.07]	1.01 [0.98, 1.05]	0.99 [0.94, 1.03]	0.95 [0.93, 0.98] ***	0.95 [0.92, 0.98] ***
Educational level (ref = JSS)								
Senior Secondary	0.90 [0.44, 1.83]	0.65 [0.29, 1.44]	0.56 [0.38, 0.93] *	0.60 [0.35, 1.03]	1.01 [0.92, 3.50]	1.06 [0.89, 3.40]	1.31 [0.95, 1.80]	1.46 [0.98, 2.05]
Diploma/Tertiary	0.46 [0.10, 2.12]	1.12 [0.17, 7.37]	0.24 [0.08, 0.69] **	0.25 [0.08, 0.77] *	1.59 [0.67, 3.81]	1.48 [0.60, 3.64]	0.85 [0.50, 1.44]	1.09 [0.62, 1.99]
Married	0.47 [0.20, 1.12]	0.68 [0.27, 1.74]	1.93 [0.87, 4.30]	1.14 [0.59, 3.20]	1.66 [0.78, 3.52]	1.82 [0.82, 4.04]	0.60 [0.37, 0.85] **	0.53 [0.34, 0.82] **
Permanent employment status	0.40 [0.20, 0.79] **	0.35 [0.17, 0.71] **	14.3 [4.6, 44.9] **	13.3 [4.19, 42.4] **	0.52 [0.30, 0.90] *	0.60 [0.34, 1.06]	1.23 [0.86, 1.75]	1.23 [0.86, 1.75]
Smoking	1.49 [0.32, 6.94]	1.43 [0.30, 6.83]	0.71 [0.15, 3.36]	0.84 [0.17, 3.96]	2.19 [0.69, 6.98]	2.21 [0.69, 7.09]	0.58 [0.23, 1.48]	0.59 [0.23, 1.50]
Drink alcohol	2.65 [1.41, 5.01] **	2.35 [1.25, 4.78] *	5.05 [2.84, 8.95] ***	5.29 [3.21, 8.72] ***	0.65 [0.39, 1.08]	0.83 [0.48, 1.42]	0.88 [0.66, 1.18]	0.72 [0.52, 0.98] *
*Job and environmental exposures*								
Job category (ref = Tunnelling areas)								
Mining areas		0.54 [0.12, 1.72]		1.41 [0.70, 2.84]		1.04 [0.46, 2.31]		1.24 [0.78, 1.97]
Combining areas		0.31 [0.07, 1.45]		1.11 [0.54, 2.28]		0.87 [0.37, 2.06]		1.70 [1.06, 2.73] *
Helping areas		1.24 [0.59, 2.64]		1.00 [0.61, 1.68]		1.17 [0.64, 2.12]		1.11 [0.78, 1.56]
Managerial and others		0.92 [0.34, 2.49]		1.31 [0.70, 2.47]		1.03 [0.51, 2.07]		1.15 [0.76, 1.74]
Duration of mining		0.96 [0.91, 1.02]		1.04 [1.00, 1.07] *		0.97 [0.94, 1.01]		1.04 [1.01, 1.06] **
Work in high/low temperature conditions		-		4.62 [1.65, 12.96] **		1.99 [0.94, 4.26]		1.86 [0.98, 2.35]
Place bath after work (ref = Bathroom at workplace)		-						
Home		-		-		1.50 [0.57, 3.98]		0.74 [0.33, 1.66]
Where eat at work place (ref = Worker’s canteen)								
At the work place/Somewhere far off		1.97 [0.43, 8.90]		0.04 [0.01, 0.23] **		1.26 [0.58, 2.76]		0.93 [0.55, 1.59]

Outcome = Respiratory disease (asthma, emphysema, pneumothorax, silicosis); JSS = Junior Secondary School; * *p* < 0.05, ** *p* < 0.01, *** *p* < 0.001.
